# Editorial: Functional mechanisms at the avian gut microbiome-intestinal immunity interface and its regulation of avian physiological responses

**DOI:** 10.3389/fphys.2022.1063102

**Published:** 2022-10-24

**Authors:** Michael H. Kogut, Mariano Enrique Fernandez-Miyakawa

**Affiliations:** ^1^ Southern Plains Agricultural Research Center, USDA-ARS, College Station, TX, United States; ^2^ Area de Bacterioligc y Fisiologica. Instituto de Pathobioloigca, INTA, Buenos Aires, Argentina

**Keywords:** gut health, microbiota, mucosal immunity, poultry, physiology

Our understanding of the interface between the gut microbiome and avian host immunity is almost exclusively based on descriptive, associative studies which have not established causality. Clearly, the need to elucidate the causal relationships and the molecular mechanisms by which the gut microbiome influences the avian host immune system, both locally and systemically, is fundamental for the translational success of intestinal microbiota-based diagnostics, therapeutics, and adjunct therapies for avian immune development and function that impacts the poultry industry worldwide. The intestinal microbiome:innate immune interactome is a signaling hub that integrates environmental inputs of poultry, especially diet, with genetic and immune signals to translate the signals into host physiological responses and the regulation of microbial ecology. Based on mammalian studies, this network of interactions characterizes the interdependence between the innate immune system and the microbiota with the two systems affecting one another to orchestrate local intestinal and whole-organism physiology. As the basic tools for characterizing microbiomes are now widely accessible, the future of poultry (chickens, turkeys ducks, geese) microbiome research is to broaden the vision and approach to enhance understanding the functional mechanisms at the avian microbiome:immunity interface and its regulation of avian physiological responses. Thus, microbiome studies in poultry are at a challenging transition from descriptive studies of association towards mechanistic studies. Essential for this transition is a diversity of thinking (chemical and systems biology, metabolism, microbiology, physiology and immunology) and the development of novel approaches (assays and models).

## Heat stress and gut health

Heat stress (HS) is an ongoing concern to commercially produced poultry worldwide particularly now with the onset of global warming and climate change. Heat stress affects meat and egg quality, predominately through the impairment of gut function (feed intake, nutrient transport, dysbiosis, inflammation) that ultimately can compromise the sustainability of the poultry industry. Cao et al. reviewed the effects of heat stress on the gut-brain axis of poultry describing the effects of high ambient temperatures on not only on gut physiology, but also the dramatic effect on the microbiota and microbiota-derived metabolites and neurotransmitters on host metabolism, behavior, and health. Emami et al. presented new data proving that the adverse effects of heat stress on intestinal integrity, physiological performance, and carcass quality was independent of the reduced feed intake normally associated with heat stress. Further work done by the Dridi group (Abdelli et al.) demonstrated that heat stress altered nutrient transporters in the intestine of different modern broiler breeds and their ancestral jungle fowl. Interestingly, the phenotype differences in growth between lines (slow, moderate, rapid) appear to play a role in the susceptibility of the lines to the adverse effects of heat stress. Lastly, two submissions by the Jha group in Hawaii (Liu et al.; Liu et al.) reported the profound negative effects of heat stress on the intestinal physiology, barrier function, immunity, the microbiome and metabolome of the slow-growing yellow-feather broiler line indigenous to China. Many of the negative effects appear to be dependent upon the increased production of pro-inflammatory cytokine-induced microbiota dysbiosis.

## Gut microbiota and intestinal immune development

Rodrigues et al. demonstrated that activation of the immune response of chicks at- or immediately post-hatch increased the proficiency of the neonatal avian immune system to sense and react to pathogens. Especially enlightening was that neonatal immune function was dependent on specific microbiota (lactic acid bacteria) exposure on the day-of-hatch when compared to exposure at 10-day post-hatch.

## Gut health and developmentof a natural subclinical ne model

Current models of NE involve pre-exposure to disease risk factors, in combination with exogenous *C. perfringens* inoculation. He and colleagues described the development of a new model based on the natural uptake of *C. perfringens* presented in the housing environment by the chicken. This group incorporated multiple NE-associated predisposing factors to promote the natural development of Clostridium *perfringens* without inoculation and successfully reproduced subclinical NE infections.

## Alternatives to antibiotics on gut health under field conditions

The development of antibiotic-free poultry production requires the identification and characterization of defined feed additives that increase the functional components of gut health (nutrient digestion and absorption, metabolism and energy generation, a stable microbiome, mucus layer development, barrier function, and mucosal immune responses) under field conditions. Further, ability to target alternatives to antibiotic growth promoters (ATA) to specific gut compartments is of upmost importance, especially under field conditions. Bortoluzzi et al. reported that encapsulating organic acids and essential oils assured their locating to the cecum which resulted in the decease in intestinal inflammation and the maintenance of the microbiota composition similar to that observed with AGPs only; thus, demonstrating the potential of encapsulating ATAs for improved intestinal health and integrity under field conditions.

## Host-microbiota interactions and hologenomics

To meet sustainability issues associated with changing environmental conditions worldwide that can affect poultry health and welfare, Tous et al. provided insights into a novel technology called hologenomics. Hologenomics provides a multi-omics platform that moves from the traditional trial and error approach to a knowledge-based strategy in which understanding the biological processes that underlie the administration of feeds, feed additives or pharmaceuticals and the observed interindividual variation is prioritized. This platform, albeit with large data sets, allows provides the interpretational understanding of how host genomic features, microbiota development dynamics and host-microbiota interactions shape animal welfare and performance.

